# Identification of cuproptosis related subtypes and construction of prognostic signature in gastric cancer

**DOI:** 10.3389/fsurg.2022.991624

**Published:** 2023-01-06

**Authors:** Hao Dong, Shutao Zhao, Chao Zhang, Xudong Wang

**Affiliations:** Department of Gastrointestinal Nutrition and Hernia Surgery, The Second Hospital of Jilin University, Changchun, China

**Keywords:** gastric cancer, cuproptosis, molecule subtypes, tumor microenvironment, immunotherapy

## Abstract

Cuprotosis is a novel mechanism of cell death that differs from known mechanisms, which depends on mitochondrial respiration and is closely related to lipoylated components of the tricarboxylic acid (TCA) cycle. However, it is unclear whether cuprotosis-related genes (CRGs) affect the tumor microenvironment (TME) and prognosis of patients with gastric cancer. In this study, the genetic and transcriptional characteristics of CRGs in gastric cancer (GC) were analyzed, and five CRGs that were differentially expressed and correlated with the survival of patients were obtained. Two different molecular subtypes were identified according to the five CRGs. Then, we constructed a CRG_score applied to patients of any age, gender, and stage. Subsequently, we found that cluster B and a high CRG_score had a worse prognosis, fewer immune checkpoints, and higher tumor immune dysfunction and exclusion (TIDE) compared to cluster A and a low CRG_score. In addition, two subtypes and the CRG_score were closely associated with clinicopathological characteristics, human leukocyte antigens (HLAs) and TME cell infiltration. A high CRG_score was featured with decreased microsatellite instability-high (MSI-H) and mutational burden. Meanwhile, the CRG_score was significantly related to the cancer stem cell (CSC) index and chemotherapeutic response. Moreover, we developed a nomogram to predict the survival probability of patients. Our study explained the role of CRGs in GC, and the prognostic signature could potentially provide an approach for personalized tumor therapy.

## Introduction

Gastric cancer (GC), a malignant tumor with high heterogeneity, is one of the global malignant diseases. Although there are regional differences in morbidity and mortality, more than 1 million people suffer from it each year worldwide (1, 2). GC is the fourth primary cause of cancerous tumor death globally (3). The low survival rate of patients with GC is due to the fact that they are primarily in the middle and late stages (1). Common treatments for GC, such as surgery and chemotherapy, are based on traditional diagnostic measures, including clinical symptoms, imaging, and pathological data (4). However, treatments of inoperable and chemo-resistant GC patients remain many challenges needed to be addressed. Precisely personalized treatments, including immunotherapy and targeted therapy based on biomarkers such as microsatellite instability (MSI), epidermal growth factor receptor (EGFR), and programmed cell death ligand 1 (PD-L1), have been relatively novel and vital treatment strategies in recent years for those challenges (5). The purpose of them is to provide patients with more efficient and healthy drug treatments (6). Therefore, finding prognosis-related biomarkers with efficient treatment is a hot topic and an essential direction.

The self-death of normal cells could prevent cancerization to a certain extent. If damaged cells, such as DNA damage that cannot be repaired, cannot die by themselves, their genes may be mutated, which will promote the transformation to tumor cells of the offspring (7). A variety of cell death mechanisms, such as apoptosis, necroptosis, pyroptosis, autophagy, and ferroptosis, have been verified to be closely related to tumor progression (7, 8). Recently, cuproptosis is a new cell death mechanism that differed from other means. Copper is an indispensable cofactor to keep the body functioning properly. Copper accumulation can promote proteins lipidation in TCA mediated mainly by FDX1 and directly bind them, which induces loss of Fe-S cluster–containing proteins and elevation of HSP70 to activate acute proteotoxic stress leading to cell death (9). Zhang et al. revealed that ferredoxin reductase (FDXR) could regulate the expression of iron-binding protein 2 (IRP2), which affects the tumor suppressor p73, to mediate the development of tumor (10). There is a complex regulatory relationship between the tumor microenvironment (TME) and tumors. The growth of tumor cells depends on various biological factors secreted by TME. In addition to malignant cells, TME also includes adipocytes, fibroblasts, immune cells, extracellular matrix (ECM), and blood vessel-related cells (11). These components interfere with tumor progression through individual or interrelated pathways of action. For example, the vascular system provides tumor nutrition and distant metastasis channels, and tumor-associated macrophages (TAMs) protect tumors from immunosuppression. Fibroblasts can drive tumors away from their primary location (11, 12). However, there are very few studies of cuproptosis in tumors. The relationships between it and tumors, TME, and the prognosis of patients are not clear.

Due to cuprotosis as a newly discovered cell death mode, its role in gastric cancer is little known. We conducted a multifaceted analysis of cuprotosis-related genes (CRGs), hoping to discover the possible mechanism of CRG in the development of gastric cancer. This study explored CRGs' expression profile and associated transcription factors, as well as survival analysis. Then, two subtypes were identified based on CRGs, and TME cell infiltration using CIBERSORT, ssGSEA and ESTIMATE algorithms, survival time, clinical features, TIDE, immune checkpoints, and HLAs between them were analyzed. Subsequently, 567 patients with GC were again divided into two subtypes based on the differentially expressed genes (DEGs) of the two subtypes. In addition, CRG_score was constructed to predict prognosis, clinical characteristics, TME cell infiltration, TIDE, immune checkpoints, HLAs, TMB, CSC, MSI, and drug sensitivity of GC patients.

## Materials and method

### Data sets source and tissue samples

Gene expression (FPKM value), clinicopathological information, copy number variation, and nucleotide mutation data of GC were obtained from The Cancer Genome Atlas (TCGA) in January 2022. GSE15459 was downed from the Gene Expression Omnibus (GEO). FPKM values are converted to TPM values. The two datasets were merged, quantile normalized, and removed batch effects by the “Combat” algorithm. Comprehensive information of patients in TCGA and GSE15459 was shown in Supplementary Table S1. CRGs were identified from the literature (Supplementary Table S2) (9). Tumor-associated transcription factors were downloaded from the website (http://www.cistrome.org/). 10 GC cases of fresh frozen tumors and adjacent tissues from the Second Hospital of Jilin University were selected for quantitative real-time PCR (qRT-PCR).

### The analysis of subtypes for CRGs

The expression of twelve CRGs was compared between 32 normal and 375 tumor samples in TCGA. Nine CRGs were differentially expressed (*P*-value <0.05). Then the Kaplan–Meier (KM) survival analysis was performed to screen out five CRGs with survival significance (*P*-value <0.05). We identified two molecular subtypes with the package “ConsensusClusterPlus” based on the five CRGs (FDX1, DLAT, PDHA1, SLC31A1, ATP7B). Moreover, core transcription factors associated with CRGs were obtained *via* the package dplyr and the Cytoscape software, in which the filter condition was the correlation coefficient >0.45 and false discovery rate (FDR) < 0.001. The biological function differences using Gene Set Variation Analysis (GSVA) and differential expression of CRGs between the two subtypes were analyzed.

### Evaluation of clinicopathological data and TME between subtypes

To explore the clinical application, we analyzed differences in age, sex, stage, survival time, and status between the two subtypes. The survival analysis was performed by survival and survminer packages. The CIBERSORT and ssGSEA algorithms were used to calculate infiltration scores of various immune cells. We assessed the immune score, stromal score, and estimate score in distinct subtypes *via* ESTIMATE algorithm. To further study the differences in immune status between subtypes, TIDE score, dysfunction score, and immune exclusion score were checked, which have a negative correlation with patients' prognosis and immune efficacy (http://tide.dfci.harvard.edu/). To better evaluate the sensitivity of immunotherapy, we compared scores for twenty common therapeutic targets such as CTLA4, CD80, VTCN1, and LAG3 among others, between subtypes. Meanwhile, the expression of HLAs was explored.

### Identification and analysis of DEGs

We obtained DEGs between the two subtypes using the limma package (|log Foldchange (FC) | > 0.585 and FDR < 0.05). To better define the cuproptosis subtypes, we retyped all samples based on DEGs by utilizing the ConsensusClusterPlus package and identified two clusters again. Subsequently, the survival analysis, clinical information and the expression of CRGs were performed between two clusters. Furthermore, GO and KEGG were used to explore the functional pathways of DEGs.

### Construction and validation of the prognostic CRG_score

Firstly, all samples were equally divided into train and test groups. Then, samples from the train group were used to construct the prognostic CRG_score. Univariate Cox regression analysis was utilized to find prognostic-related DEGs. Next, LASSO regression analysis for prognostic-related DEGs using the glmnet package in R was conducted to prevent overfitting. Subsequently, the seven genes and formula of CRG_score (e^(each gene's expression × correlative coefficient)^) were obtained using the multifactorial Cox analysis. According to this formula, each sample obtained a risk score. Samples of the train group were divided into high- and low CRG_score groups based on the median risk score. The test group and all sets were divided into two groups *via* the same method. The survival ROC package was used to construct the ROC curves and obtain the area under the ROC curve (AUC) for 1, 3, 5, and 7 year OS, which can check the accuracy of the model. Moreover, survival time, clinical information, TME cell infiltration, TIDE, immune checkpoints, and HLA were analyzed by the same methods as before between high- and low CRG_score groups. The Gene set enrichment analysis (GSEA) software was conducted to explore the functional pathways in two groups. The filter criterion according to the c2.cp.kegg.v7.5.1symbols.gmt gene set was |normalized enrichment score (NES)| > 1.5, nominal (NOM) *p*-value <0.05 and FDR *q*-value <0.05.

### Mutation and cancer stem cell (CSC) index analyses

The mutation data was downed from TCGA, which was analyzed to observe the distinction in high- and low CRG_score groups by the maftools package. Meanwhile, we studied the relationships of TMB with survival, the two CRG_score groups and risk score. At the same time, the correlation of TMB with immune cells was explored. We also performed a correlation analysis of CSC and risk score.

### Microsatellite instability (MSI) and drug susceptibility analyses

MSI could be used to guide clinical medication. Therefore, the relation of MSI with risk score was studied. To guide the clinical application of drugs, we screened out the common chemotherapeutic drugs based on differential half inhibitory concentration (IC50) values between the two CRG_score groups using pRRophetic and limma packages.

### Construction of a nomogram

A nomogram was constructed using the rms package to predict the patients' 1, 3, 5 and 7 -year survival probability, while the calibration curve examined the nomogram's forecasting performance.

### Quantitative real-time PCR

Total RNA was extracted from GC patient tissues using Trizol reagent (Invitrogen, Carlsbad, CA, United States). A reverse transcription kit (Takara, Tokyo, Japan) was used to synthesize cDNA. The SYBR Premix Ex Taq™ kit (Takara, Japan) was used to perform the RT-qPCR. The mRNA expression level of SLC25A15, CTSV, RGS4, SYT13, ENTPD2, CA8, and NPTX1 was normalized by GAPDH. The data were determined by the −ΔΔCt means. The primers of the seven genes were listed in Supplementary Table S3.

### Statistical analyses

All statistical analyses were conducted by R version 4.1.1. *P* < 0.05 was considered statistically significant.

## Results

### Genetic and transcriptional characteristics of CRGs in GC

**Figure 1:**
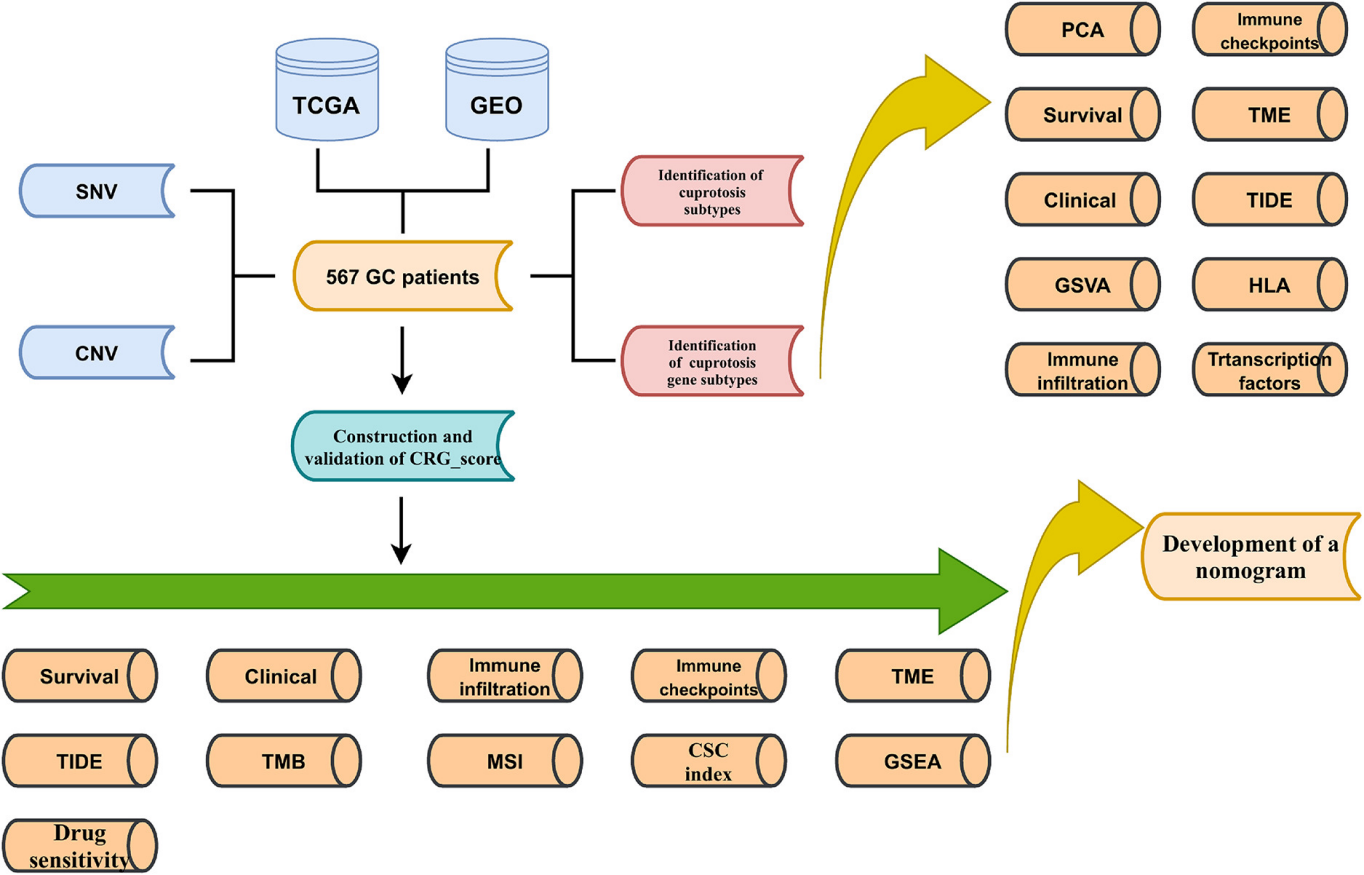
Schematic diagram of the flow of the study.

**Figure 2:**
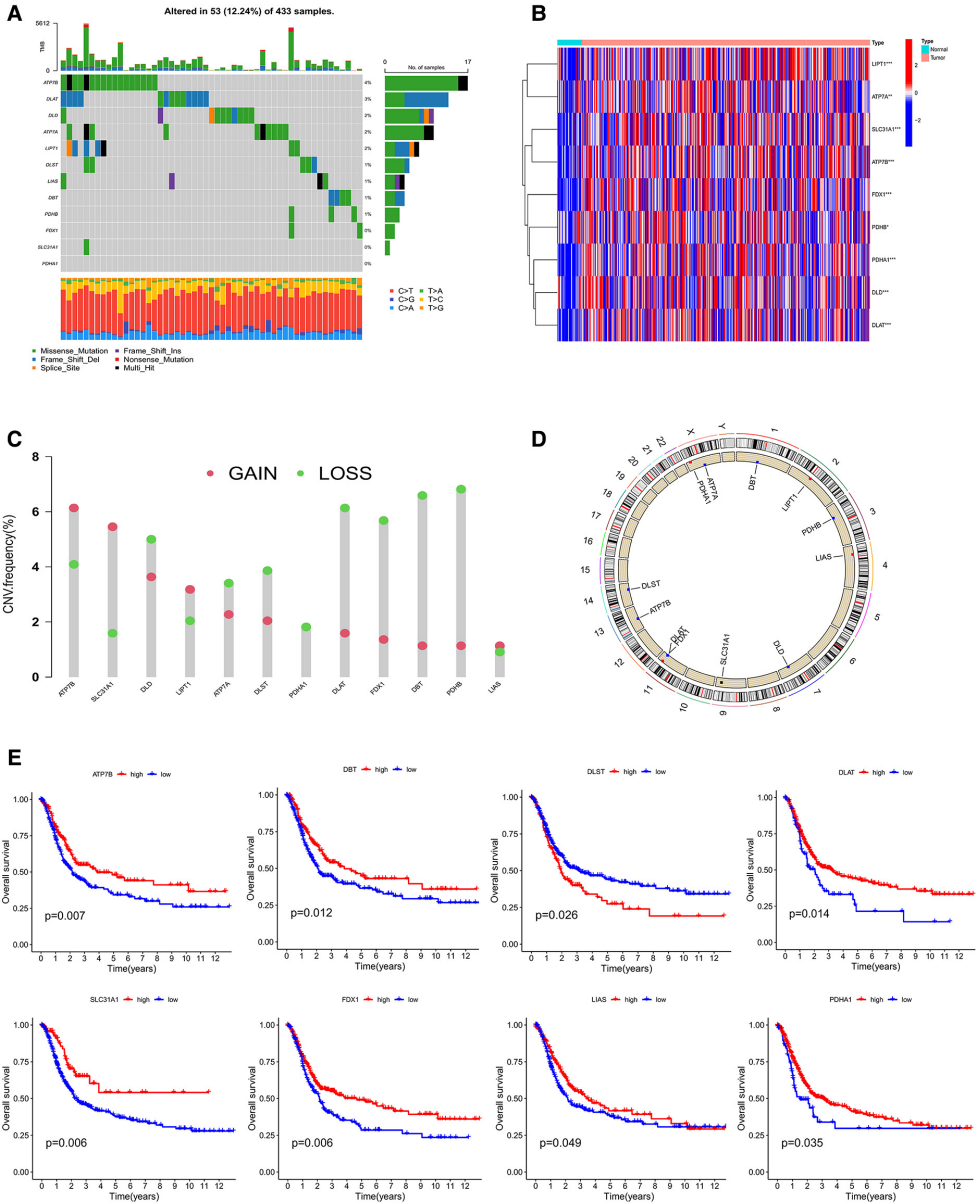
(**A**) mutation characteristic of 12 CRGs from TCGA. (**B**) The expression of CRGs between Normal and tumor. (**C,D**) The changes of somatic copy number variation in CRGs. (**E**) The survival analysis of CRGs. CRGs, cuprotosis-related genes. (*P < 0.05; **P < 0.01; ***P < 0.001; ns, not significant).

The overall experimental design was presented in Figure 1. First, the mutation status in 12 CRGs were explored (Figure 2A). The overall mutation rate is 12% (53/433 samples). ATP7B, DLAT, DLD, ATP7A, and LIPT1 were the top five genes with relatively high levels of mutations. Then, the analyses of the correlation between CRGs expression and mutations were performed. Mutants tended to be accompanied by increased expression of CRGs (DBT, LIAS, DLAT, DLD, PDHB, especially LIAS) compared to wild-type (Supplementary Figure S1). 9 CRGs (75%, 9/12 CRGs) are differentially expressed between normal and tumor tissues (Figure 2B). Almost all 12 CRGs had the changes of somatic copy number variation (CNV). The CNV increases happened in ATP7B, SLC31A1, and LIPT1; nevertheless, DLAT, FDX1, DBT, and PDHB occurred the CNV decreases (Figure 2C). Their chromosomal sites were described in Figure 2D. We performed the survival analyses on 567 samples with GC based on the expression of CRGs. And we observed that 8 CRGs were significant (*P *< 0.05). Among them, seven highly expressed CRGs (87.5%) had a good prognosis, which suggested CRGs may have inhibitory effects on gastric cancer (Figure 2E).

### Identification of cuproptosis subtypes in GC

**Figure 3:**
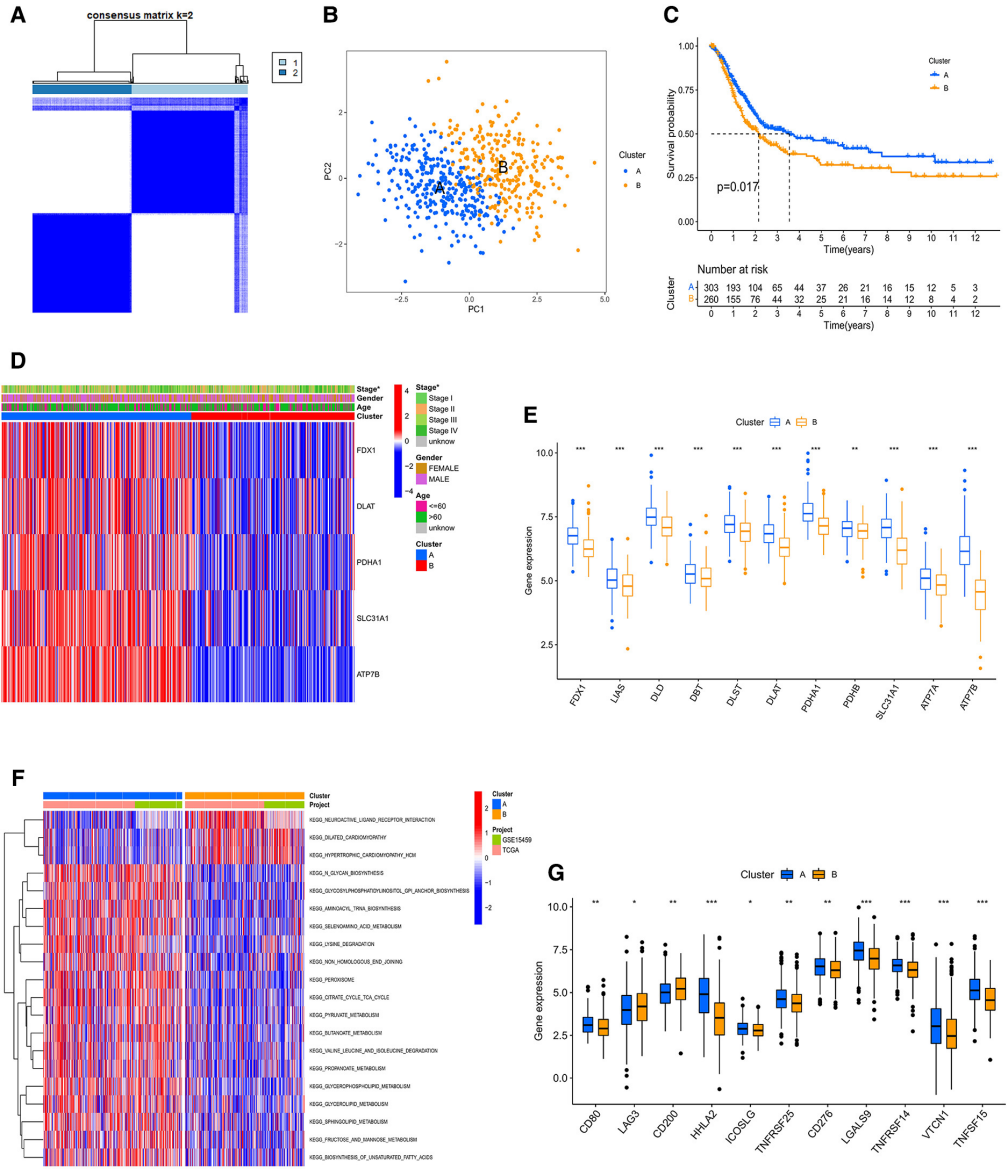
(**A**) consensus clustering matrix defining two clusters (*k* = 2). (**B**) The PCA analysis of the two clusters describing an obvious difference. (**C**) The KM curve between A and B subtypes. (**D**) The heatmap showing the correlation of the two subtypes with age, stage, gender, and CRGs. (**E**) The mRNA expression of CRGs between A and B subtypes. PPI, Protein-protein interaction; KM, Kaplan–Meier. (**F**) GSVA of analysis between two distinct subtypes, in which red represents positively correlated pathways and blue negatively correlated pathways. (**G**) Differential expression of immune checkpoints in the two subtypes.

To study the classification of cuproptosis in GC, the 567 samples were classified *via* the consensus clustering analysis according to the expression profiles of 5 CRGs. There were A (*n* = 305) and B (*n* = 262) subtypes in terms of the best *k* value (*k* = 2) (Figure 3A and Supplementary Figure S2A). The rationality of the clusters was further verified by PCA analysis (Figure 3B). The survival analysis demonstrated that subtype A had a better prognosis than B (Figure 3C). For clinicopathological features, stages of cluster A were lower compared to B (Figure 3D). At the same time, we further analyzed the expression of CRGs of clusters to explore the reasons for differences between clusters. The results indicated that 11 CRGs (91.6%) were more highly expressed in cluster A than B (Figure 3E). Moreover, GSVA proved that cluster A was enriched in N-glycan biosynthesis, citrate and TCA cycles, the metabolisms of sphingolipid, glycerolipid, propanoate, butanoate, pyruvate, fructose and mannose, and cluster B was enriched in neuroactive ligand-receptor interaction (Figure 3F and Supplementary Table S4). At the same time, we evaluated the expression of 20 immune checkpoints between cluster A and B, which showed that 11 checkpoints were differential, of which 9 checkpoints (CD80, HHLA2, ICOSLG, TNFRSF25, CD276, LGALS9, TNFRSF14, VTCN1, TNFSG15) had higher expression of cluster A than B (Figure 3G). Cross-metabolic reprogramming of cancer and immune cells is seen as a determinant of the antitumor immune response. More and more studies have shown that cancer metabolism could regulate antitumor immune response by releasing metabolites. Moreover, immune cells also undergo metabolic reprogramming during proliferation, differentiation, and effector function (13). Results of GSVA and GO analysis mainly focus on cancer metabolism, which is closely related to tumor immune response. In fact, tumor immune response has long been recognized as an important factor in the efficacy of immunotherapy and the prognosis of cancer patients. Therefore, we mainly analyzed the results related to tumor immune infiltration.

### Characteristics of TME cell infiltration, TIDE and checkpoints in the cuproptosis subtypes

**Figure 4:**
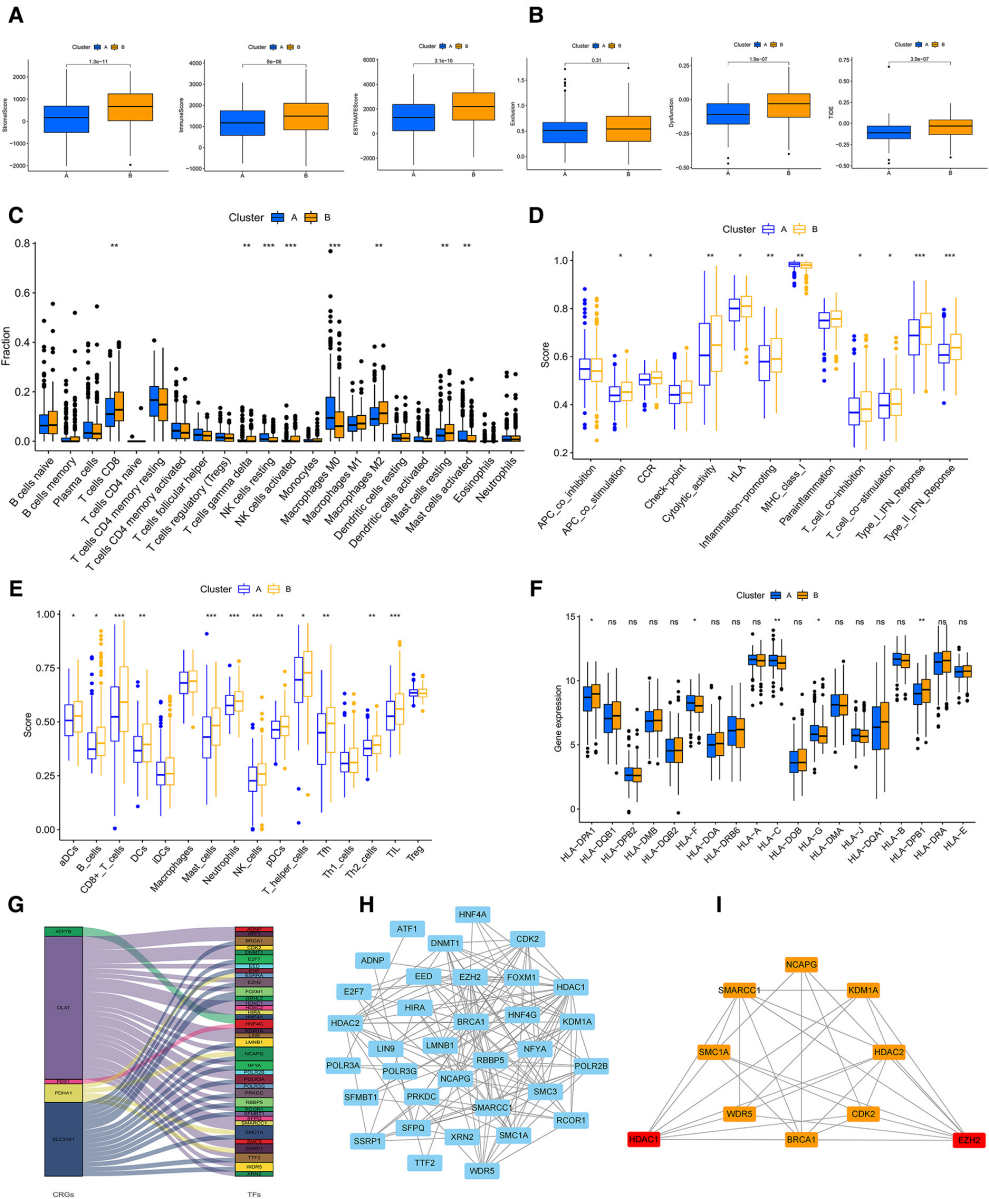
(**A**) The stromal, immune, ESTMATE scores of A and B subtypes using ESTMATE algorithm. (**B**) Associations of the TIDE score and the two subtypes. (**C**) TME cells infiltration in A and B subtypes by CIBERSORT algorithm. **(D,E**) ssGSEA algorithm calculating the scores of immune cells infiltration in the two clusters. (**F**) The plot showing the expression of HLAs in the two clusters. (**G**) Sankey diagram of CRGs and transcription factors. (**H**) PPI network of the CRGs based on the STRING database. (**I**) The plot of hub transcription factors visualized by Cytoscape. GSVA, gene set variation analysis; TIDE, Tumour Immune Dysfunction and Exclusion; HLAs, human leukocyte antigens; TME, tumor microenvironment. (*P < 0.05; **P < 0.01; ***P < 0.001; ns, not significant).

In order to comprehensively analyze the relationship between CRGs and TME in GC, we observed that stromal, immune, and estimate scores of cluster B were higher than cluster A using the ESTIMATE algorithm (Figure 4A). The scores of dysfunction and TIDE were lower in cluster A than B (Figure 4B). We used CIBERSORT, ssGSEA, and ESTIMATE algorithms to evaluate. The results of CIBERSORT algorithm demonstrated that the differences between cluster A and B were concentrated in T cells, NK cells, Macrophages, and Mast cells (Figure 4C and Supplementary Table S5). The ssGSEA algorithm also verified similar outcomes for these cells (Figures 4D,E and Supplementary Table S6). In addition, For HLA, cluster A had lower expression of DPA1 and DPB1 than B, and the opposite for HLA C and G (Figure 4F). Prognosis-related and differentially expressed 5 CRGs were selected to study their transcription factors, which were demonstrated in Figures 4G,H. Ten hub genes, especially HDAC1 and EZH2 were screened by the Cytoscape software (Figure 4I). Moreover, the expression of core transcription factors in subtype A was higher than that in subtype B (Supplementary Figure S2B).

### Identification and analysis of gene subtypes based on DEGs

**Figure 5:**
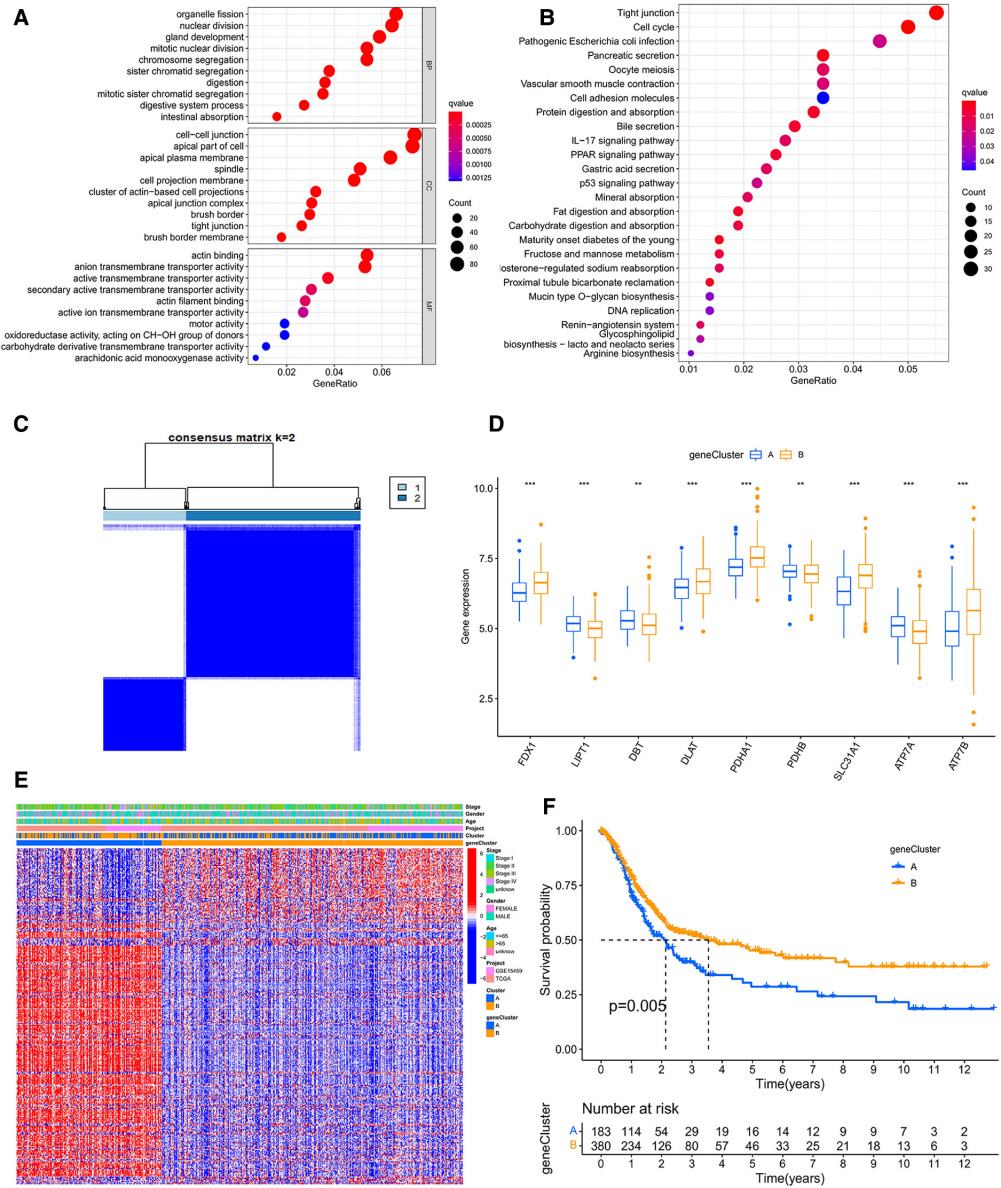
(**A**) The GO analysis based on the differentially expressed genes (DEGs). (**B**) The KEGG analysis of DEGs. (**C**) Consensus clustering matrix defining two clusters (*k* = 2) accroding to DEGs. (**D**) The expression of CRGs between A and B geneclusters. (**E**) The heatmap showing the correlation of the subtypes and geneclusters with clinicopathological data and CRGs. (**F**) The survival curve between A and B geneclusters. GO, Gene Ontology; KEGG, Kyoto Encyclopedia of Genes and Genomes.

In order to identify the cuproptosis subtypes clearly, we screened 1,233 DEGs between the two cuproptosis subtypes through the limma package. GO and KEGG for these DEGs were enriched mainly in cell cycle, fat digestion and absorption, PPAR signaling pathway, IL-17 signaling pathway, p53 signaling pathway and DNA replication, which were closely related to cancer (Figures 5A,B and Supplementary Table S7). Furtherly, we used the univariate COX method to select 304 prognostic-related DEGs and classified all samples according to them (Supplementary Table S8). The results showed that there were still two gene subtypes, which was consistent with the previous typing results (Figure 5C and Supplementary Figure S2C). There were significant differences in expression of CRGs, clinical traits and survival time between A and B gene clusters (Figures 5D, E, F).

### Construction of the prognostic CRG_score

LASSO regression analysis was performed for 304 prognostic-related DEGs. We chose the lambda minimum to select the appropriate genes (Supplementary Figure S2D,E). Subsequently, seven genes (SLC25A15, CTSV, RGS4, SYT13, ENTPD2, CA8, NPTX1) were screened to construct the model by the multifactorial Cox analysis. The genes included four high-risk genes (CTSV, RGS4, SYT13, NPTX1) and three low-risk genes (SLC25A15, ENTPD2, CA8). The formula of CRG_score was shown as follows:

Risk score = (−0.27565756734421* expression of SLC25A15) + (−0.235708601387405* expression of ENTPD2) + (−0.124572834187939* expression of CA8) + (0.379774295825035* expression of CTSV) + (0.212171866831215* expression of RGS4) + (0.160140827663568* expression of SYT13) + (0.128438559010339* expression of NPTX1).

**Figure 6:**
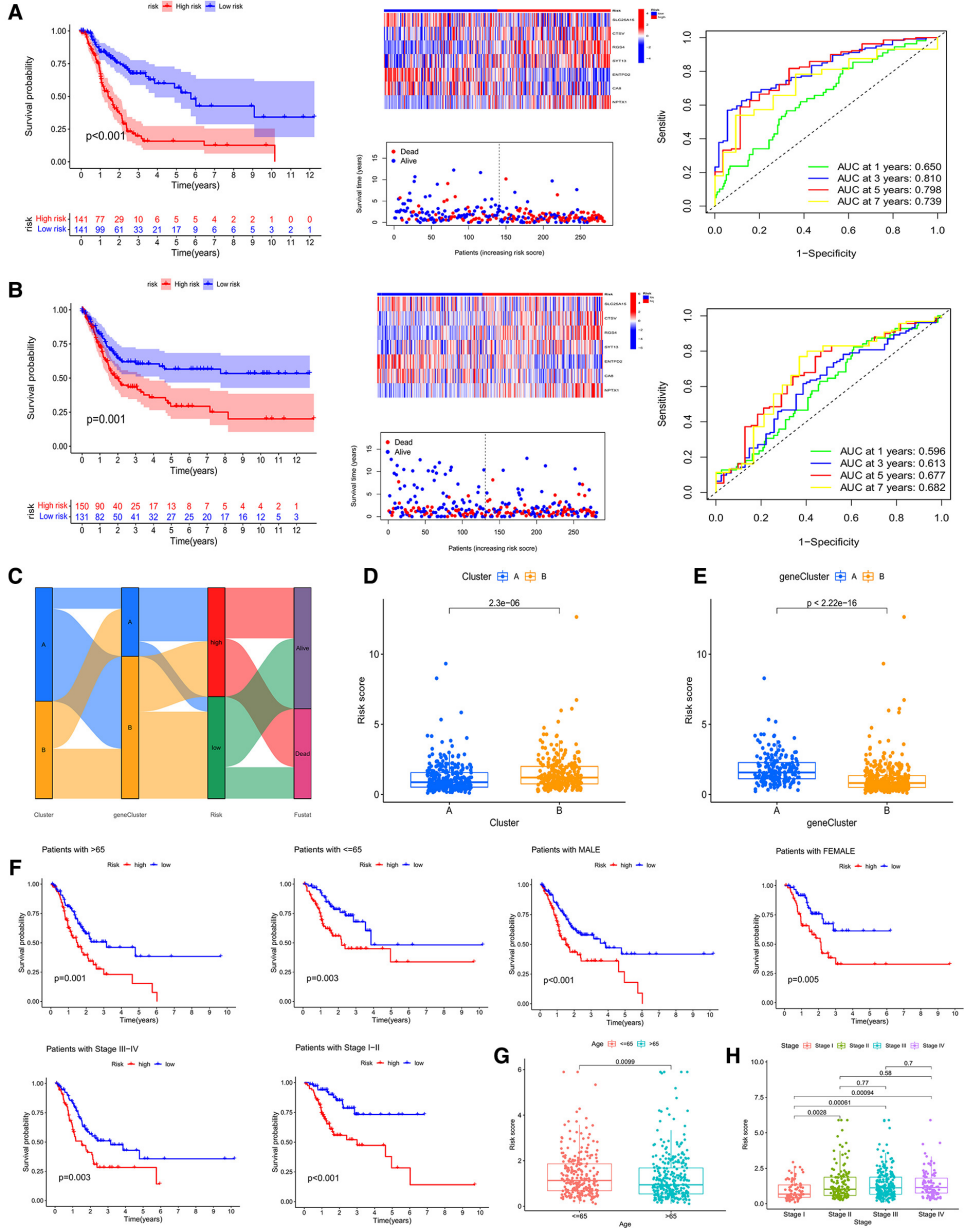
survival analysis, heatmap, survival status accompanied with the CRG_score and ROC analysis in the train cohort (**A**) and the test cohort (**B**). (**C**) Sankey picture showing the relationship of subtypes, geneclusters, risk groups and patients survival status. (**D,E**) Correlation of two subtypes and genecluster and CRG_score, respectively. (**F**) Applicability of CRG_score to GC patients of any age, gender and stage. (**G,H**) Relationship of age and stage with CRG_score, respectively.

Then, both the train group and the test group were divided into high- and low- CRG_score groups. The survival curve demonstrated that the survival of low- CRG_score group was better than high-CRG_score group in the train group (*P *< 0.001). The AUC values of 1-, 3-, 5-, and 7- year were 0.650, 0.810, 0.798, and 0.739, respectively (Figure 6A). For the test group, the result of survival analysis was the same of the train group. The AUC values of 1-, 3-, 5-, and 7- year were 0.596, 0.613, 0.677, and 0.682, respectively (Figure 6B). The relationships between cuproptosis subtypes, gene subtypes, CRG_score groups, and patients' survival status were shown in Figure 6C. Meanwhile, cluster A was associated with a low score, which was consistent with previous survival analyses of both subtypes and CRG_score groups (Figure 6D). Gene cluster A was associated with a high score and low survival time (Figure 6E). For gastric cancer patients of any age, gender, and stage, a high CRG_score was accompanied by low survival time (Figure 6F). There was a significant correlation between the age of the patient and the score (Figure 6G). Stage I patients had a lower score than stage II, III, and IV (Figure 6H).

### Correlation of TME cell infiltration, TIDE and checkpoints with high- and low-CRG_score groups

**Figure 7:**
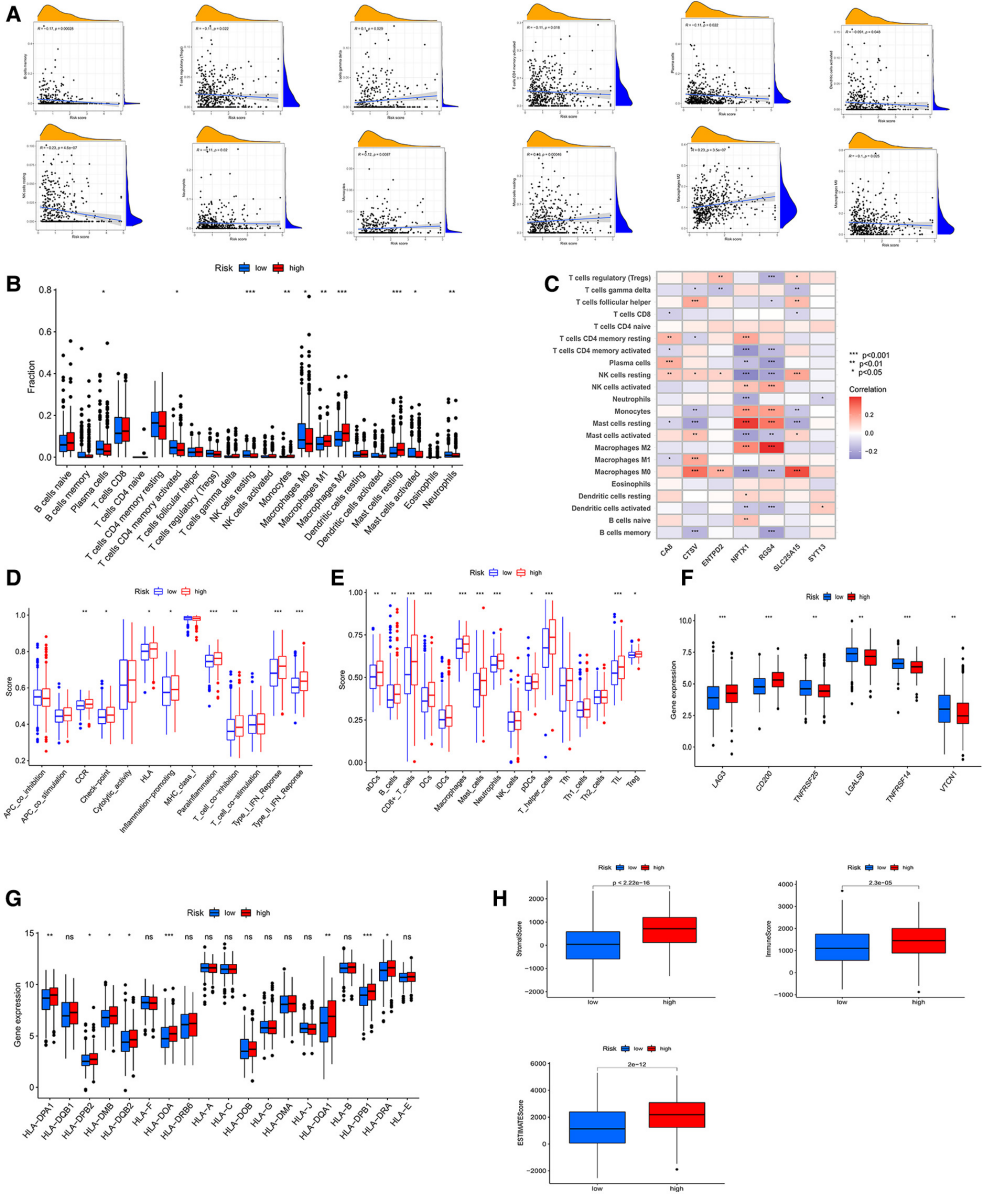
(**A**) correlative analysis between risk score of immune cells. (**B**) TME cells infiltration in low- and high-CRG_score groups by CIBERSORT algorithm. (**C**) Associative analysis between 22 immune cells and risk genes. (**D,E**) ssGSEA algorithm calculating the scores of immune cells infiltration in the risk groups. (**F**) Differential expression of immune checkpoints in the two CRG_score groups. (**G**) The diagram showing the expression of HLAs in the two risk groups. (**H**) The stromal, immune, ESTMATE scores of low- and high-CRG_score groups using ESTMATE algorithm.

**Figure 8:**
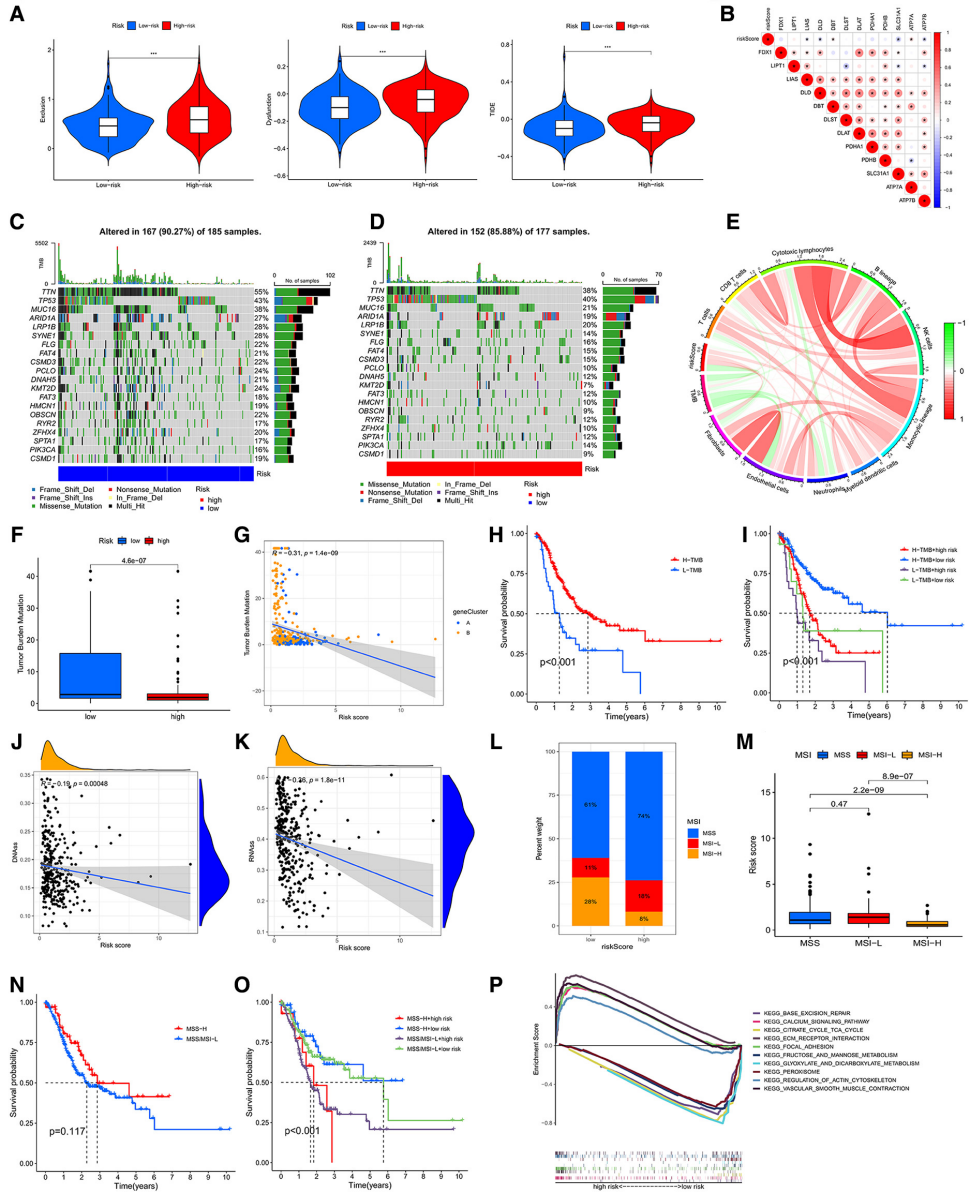
(**A**) associations of the TIDE score and the two risk groups. (**B**) Correlative analysis between risk score and expression of CRGs. Waterfall chart showing the top 20 mutated genes and their frequencies in the low-risk group (**C**) and the high-risk group (**D**). (**E**) Circle graph showing the relationship between TMB, immune cells and risk score. (**F**) The diagram showing the levels of TMB in the two risk groups. (**G**) Correlative analysis between risk score and expression of TMB. (**H**) The survival analysis of the low- and high-TMB groups. (**I**) The survival analyses of combined TMB and risk groups. (**J,K**) Cancer stem cell (CSC) index analyses of riskscore. (**L,M**) Associative analysis between MSI and riskscore. (**H**) The survival curve of the MSS/MSI-L and MSI-H groups. (**O**) The survival analyses of combined MSI and risk groups. (**P**) The GSEA analysis of low- and high-CRG_score groups.

Similarly, we used three algorithms to assess TME cell infiltration between high- and low-CRG_score groups. By CIBERSORT, we found that low CRG_score was accompanied by high B cells memory, Tregs, T cells CD4 memory activated, plasma cells, NK cells resting, neutrophils, mast cells activated, dendritic cells activated, and Macrophages M0, and low T cells gamma delta, monocytes, mast cells resting, Macrophages M2 (Figures 7A,B). Meanwhile, the outcomes of the correlation of TME cell infiltration with risk genes were described in Figure 7C. The relationships of TME cell infiltration with survival time were shown in Supplementary Figures S2F,G. The analysis of ssGSEA also suggested a significant correlation between TME and two CRG_score groups (Figures 7D,E). Expressions of 20 immune checkpoints were assessed between high- and low-CRG_score groups, which showed that 6 checkpoints were differential, of which 4 checkpoints (TNFRSF25, LGALS9, TNFRSF14, VTCN1) had higher expression of low- than high-CRG_score group (Figure 7F). For HLA, the low-CRG_score had lower expression of DPA1, DPB2, DMB, DQB2, DOA, DQA1, DPB1 and DRA than the high-CRG_score (Figure 7G). At the same time, we found that stromal, immune, and estimate scores of the high-CRG_score group were higher than the low-CRG_score group through the ESTIMATE algorithm (Figure 7H). The high CRG_score had the high scores for dysfunction, exclusion and TIDE (Figure 8A). The correlation analysis of risk scores and CRGs was described in Figure 8B.

### Mutation and CSC index analysis

Accumulation of mutations in somatic cells can cause their transformation into cancer cells (14). Meanwhile, a study showed a positive correlation between high TMB and better survival in many cancers (15). Therefore, we downloaded and analyzed the data of mutation from TCGA. The top 10 mutated genes in the low-CRG_score group were TTN (55%), TP53 (43%), MUC16 (38%), ARID1A (27%), LRP1B (28%), SYNE1 (28%), FLG (22%), FAT4 (21%), CSMD3 (22%), PCLO (24%) (Figure 8C). The top 10 genes mutated in the high-CRG_score group were the same as those genes, but the rates of mutation were low than low-CRG_score group (38%, 40%, 21%, 19%, 20%, 14%, 16%, 15%, 10%, 12%, correspondingly) (Figure 8D). TMB might have associations with endothelial cells, neutrophils, cytotoxic lymphocytes and B lineage (Figure 8E). Similarly, the low CRG_score had a high TMB (Figure 8F). The relationship between TMB and CRG_score was plotted in Figure 8G. Moreover, our results also testified that high TMB had a good prognosis than low TMB (Figure 8H). A combined analysis of risk score and TMB demonstrated that H-TMB + low risk had the best prognosis, but L-TMB + high risk had the worst prognosis (Figure 8I). There was a negative association between CSC index and risk score (DNAss: *R* = −0.19, *P *< 0.001; RNAss: *R* = −0.36, *P *< 0.001) (Figures 8J,K).

### MSI, GSEA and drug susceptibility analysis

**Figure 9:**
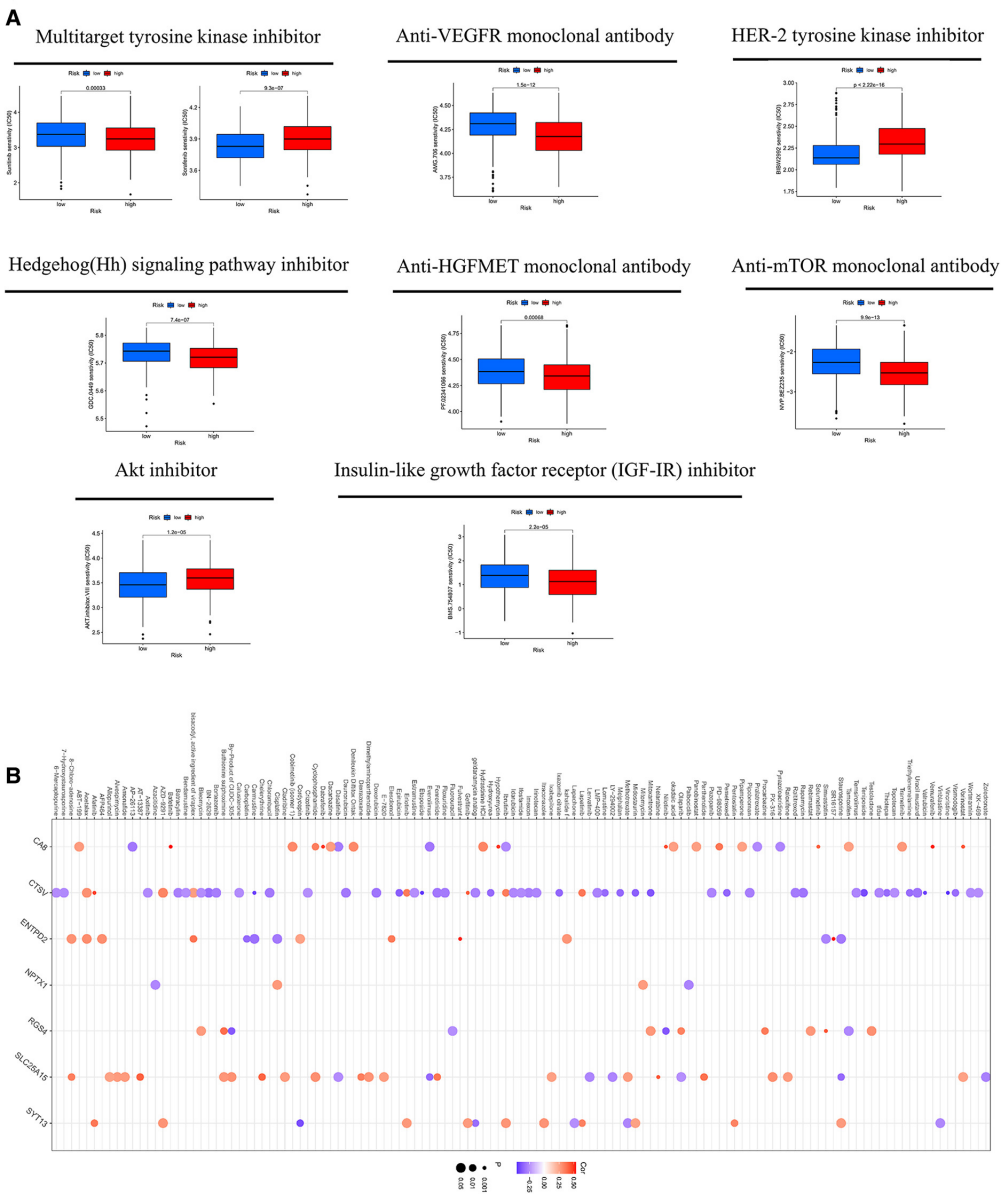
(**A**) the sensitivity of eight types of drugs between high- and low-CRG_score groups. (**B**) The sensitivity of chemotherapeutic drugs to risk genes.

MSI may predict the efficacy of chemotherapy and immunotherapy and survival of patients (16). Our study suggested that the risk score of MSI-H was higher than MSS and MSI-L (Figures 8L,M). The survival analysis of MSI-H and MSS/MSI-L was not meaningful (Figure 8N). A combined analysis of risk score and MSI proved that MSI-H + low risk had the best prognosis, but MSS/MSI-L + high risk had the worst prognosis (Figure 8O). The GSEA analysis manifested that the high-CRG_score group was fastened on base excision repair, calcium signaling pathway, citrate and TCA cycle, ECM receptor interaction, and focal adhesion. The low-CRG_score group was concentrated on fructose and mannose metabolism, glyoxylate and dicarboxylate metabolism, peroxisome, regulation of actin cytoskeleton, and vascular smooth muscle contraction (Figure 8P). To evaluate the difference of drugs between the two risk groups, we screened 67 drugs using the pRRophetic package, of which 13 drugs had higher IC50 values in the high-risk group than in the low-risk group. And the remaining 54 drugs were the opposite (Figure 9A and Supplementary Figures S3, S4). Furthermore, we screened eight types of drugs which were commonly used in GC according to the results of all drugs, including multitarget tyrosine kinase inhibitor, anti-VEGFR monoclonal antibody, HER-2 tyrosine kinase inhibitor, Hedgehog(Hh) signaling pathway inhibitor, anti-HGFMET monoclonal antibody, anti-mTOR monoclonal antibody, Akt inhibitor, Insulin-like growth factor receptor (IGF-IR) inhibitor. The results showed that sensitivities of sunitinib, AMG.706, GDC.0449, PF.02341066, BMS.754807 for GC patients in the high-risk group were higher than the low-risk group; sorafenib, BIBW2992 and NVP.BEZ235 were opposite (Figure 9A). At the same time, we also analyzed a variety of chemotherapeutic drugs that could be sensitive to risk genes (Figure 9B).

### Construction and validation of a nomogram

**Figure 10:**
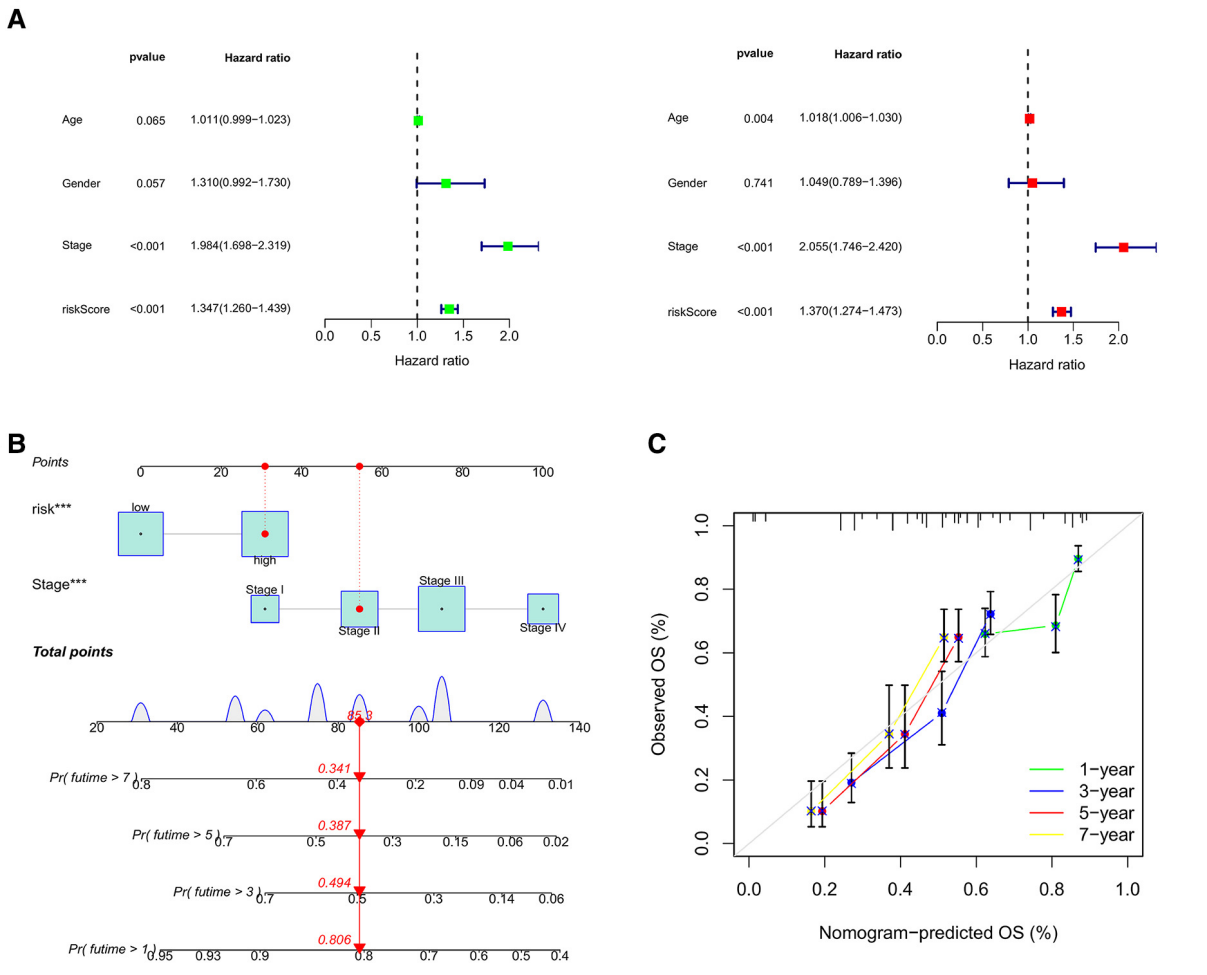
(**A**) the forest illustrations displaying univariate and multifactor COX regression analysis. (**B**) The nomogram to predict the survival times of 1, 3, 5, and 7 years. (**C**) The calibration graphs of 1, 3, 5, and 7 years.

Using univariate and multifactor COX regression analysis, we discovered that CRG_score and stage were independent prognostic factors in gastric cancer patients (Figure 10A and Supplementary Figures S2H,I). Therefore, CRG_score and stage were used to develop a nomogram to predict the survival times of 1, 3, 5, and 7 years (Figure 10B). The calibration graph displayed that the predictive ability of the nomogram was relatively accurate (Figure 10C).

## Discussion

Copper, one of the most vitally basic trace metals in the human body, involves in various biological functions, including the regulation of enzyme function, cofactor for growth and development, redox processes, energy metabolism, iron absorption and cell proliferation (17). Like iron, copper is also closely related to the development of cancers. On the one hand, copper can promote tumorigenesis by activating the MAPK pathway (18). Copper-binding enzymes mediate HIF1-*α* and Snail to promote the Epithelial to Mesenchymal Transition (EMT) progression of tumors (19). The copper-enzymes LOX (Lysyl Oxidase) promotes adhesion and metastasis of colorectal cancer by affecting the turnover of the Extracellular Matrix (ECM) (20). On the other hand, copper can indirectly suppress tumors by promoting alterations in the recruitment of myeloid precursors or affecting tumor-associated macrophages (TAMs) (17). Accumulation of copper can activate oxidative stress leading to tumor cell death, which may be a potential treatment for cancer (21). Therefore, the role of copper in cancer is complex. In this study, we observed that the expression of CRGs in GC was higher than in normal samples, which was the same as the previous report that copper was elevated in many tumor tissues (22, 23). However, the survival analysis of 567 GC patients indicated that high expression of most CRGs had a better prognosis than low expression. This suggested that the high expression of CRGs may inhibit tumor cells in some way. Recently, Peter Tsvetkov et al. found that the accumulation of copper can induce cell death in a novel mechanism, namely cuproptosis (9). This finding enlightened us that CRGs may induce gastric cancer cell death through cuproptosis, thereby improving the prognosis of patients. Therefore, based on CRGs, we identified two subtypes and developed a CRG_score to explore the role of CRGs in gastric cancer comprehensively.

In our study, we testified that subtype A had a lower CRG_score than B, which suggested subtype A seemly was accossicated with the low-CRG_score group. Meanwhile, we discovered that the survival times and clinical stages of subtype A and the low CRG_score group were lower than subtype B and the high-risk group. Our findings also demonstrated that subtype A and the low CRG_score group had high expression of CRGs. These results again confirmed that high expression of CRGs was related to a better prognosis. Subsequently, ssGSEA and CIBERSORT algorithms showed that the differences of TME in two subtypes and CRG_score groups were Macrophages, NK cells, mast cells and T cells CD4. It is well known that macrophages can be polarized into M1 and M2 types (24). M1 type is mainly involved in the activation of the inflammatory response, while M2 is mainly involved in tissue repair and inhibition of inflammation (25, 26). Initially, macrophages are polarized to the M1 type, and cooperate with other immune cells to eliminate tumor cells. When tumor cells are in low oxygen and low pH, they will release Neuropilin-1 (Nrp-1), TGF-β, IL6, IL4, Tim-3 to promote the transformation of macrophages into M2 type, which can help tumor cells to escape immune and secrete growth factors to enhance tumor growth (27–30). Interestingly, M2 infiltrated highly in the high CRG_score group and subtype B. In the TME, inflammatory and cytotoxic effector functions of NK cells are weakened by a number of cytokines such as IL-23 and IL-1β, where NK cells are called tumor-infiltrating natural killer cells (TINKs). In addition to decreasing the ability of cytotoxicity, TINKs can also inhibit the growth and spread of T cells to reduce their damage to tumors (31, 32). Mast cells are one of the important innate immune cells in the immune system (33). Many studies have shown that low levels of mast cells were associated with poor survival and advanced tumors (34–36). Fortunately, we discovered that the activity of NK cells remained low in subtype A and low CRG_score group; however, the activity of mast cells was high. CD4+ memory T cells are important modulatory elements of the immune system (37). A study has proved that the more CD4+ memory T cells infiltrated in gastric cancer, the longer the patients' survival time (38). Tumor specific antibodies are generated by Plasma cells to damage tumor cells (39). Coincidentally, Plasma cells and CD4+ memory T cells activated were higher in the low-CRG_score than the high- CRG_score group. In summary, TME cell infiltration in subtype A and the low-risk group was more tumor suppression, which may also be one of the reasons for the better prognosis of patients in these two groups.

In this paper, GSEA enrichment analysis showed that high CRG_score was mainly enriched in base excision repair, calcium signaling pathway, citrate, and TCA cycle, ECM receptor interaction, and focal adhesion. Low CRG_score was mainly enriched in pathways related to metabolism. In conclusion, the pathways described above may be the potential mechanism by which CRG_score affects immune infiltration in gastric cancer. Immune checkpoint blockade is considered as a promising approach to immunotherapy of cancer (40). Many immune checkpoints such as CD200, VTCN1, PD-1, CTLA-4 and LAG-3, and so on, were found (41–44). Therefore, we evaluated twenty immune checkpoints and found that subtype A and the low-CRG_score group had higher expression of these checkpoints. TIDE as a computational method is used to predict immune checkpoint blockade (ICB) response. A score based on TIDE is negatively correlated with the effects of immunotherapy (45). The score of TIDE for subtype A and the low-CRG_score group was low. TMB and MSI have emerged as major predictors of immunotherapy efficacy. High TMB and H-MSI often represent favorable immune infiltration and prognosis (46, 47). Interestingly, low CRG_score and subtype A were accompanied by high TMB and H-MSI. In addition, we also assessed the sensitivity of chemotherapeutic drugs between high- and low-CRG_score groups using the pRRophetic package. At the same time, we also analyzed a variety of chemotherapeutic drugs that could be sensitive to risk genes. Moreover, the results of q-RT PCR showed that CTSV, RGS4, SYT13 and NPTX1 were highly expressed in tumor tissues compared with adjacent tumor tissues, while SLC25A15, ENTPD2 and CA8 were low expressed. This is consistent with the fact that CTSV, RGS4, SYT13 and NPTX1 were high-risk genes, and SLC25A15, ENTPD2 and CA8 were low-risk genes (Supplementary Figure S5).

Transcription factors can recognize specific DNA sequences to control chromatin and transcription for directing gene expression, which constitutes a complex regulatory system (48). Many studies have shown that the changes of biological functions of transcription factors were closely related to the occurrence and development of tumors. In this paper, we found that there were significant differences in the survival time and clinical stage of patients with cuprotosis-related A and B subtypes. Therefore, we assumed whether transcription factors were one of the influencing factors, so we used Cor function to screen out transcription factors that were strongly correlated with the genes of the constructed subtypes, which the screening conditions were: Cor >0.45 and FDR <0.001. Then, six analysis methods (Betweenness, Closeness, Degree, Eigenvector, LAC) were integrated to screen out the core transcription factors using Cytoscape software. Finally, the expression of core transcription factors in subtype A was higher than that in subtype B. This suggests that transcription factors may play a role in the differences between A and B subtypes. This will help us to study the potential mechanism of CRGs in gastric cancer and explore the therapeutic strategies based on targeting transcription factors.

Our study also had several limitations. First, the data of our research was mainly based on the public database. Therefore, more basic experimental validation may be required. Second, our data was derived from TCGA and GEO databases. Thus, we still need to collect more samples to reduce statistical errors. At the same time, the mechanism of action between cuproptosis and immune cells needs to be further explored.

## Conclusion

Based on subtypes and prognostic signature of CRGs, the relationships between CRGs and the prognosis of patients, TME cell infiltration, immunotherapy, and drug sensitivities were comprehensively explored. Our study uncovered the roles of cuproptosis in gastric cancer, which could provide a new idea for cancer treatment.

## Data Availability

The datasets presented in this study can be found in online repositories. The names of the repository/repositories and accession number(s) can be found in the article/**Supplementary Material**.
