# Correction: Identification of cuproptosis related subtypes and construction of prognostic signature in gastric cancer

**DOI:** 10.3389/fsurg.2026.1809226

**Published:** 2026-03-13

**Authors:** Hao Dong, Shutao Zhao, Chao Zhang, Xudong Wang

**Affiliations:** Department of Gastrointestinal Nutrition and Hernia Surgery, The Second Hospital of Jilin University, Changchun, China

**Keywords:** gastric cancer, cuproptosis, molecule subtypes, tumor microenvironment, immunotherapy

The funder National Natural Science Foundation of China, 82203801 to Chao Zhang was erroneously omitted.

The original version of this article has been updated.

