# Supplementary figures and images for: Identification of cuproptosis related subtypes and construction of prognostic signature in gastric cancer
Source: Front Surg. 2023 Jan 6;9:991624. doi: 10.3389/fsurg.2022.991624 (PMC9852337; doi:10.3389/fsurg.2022.991624)

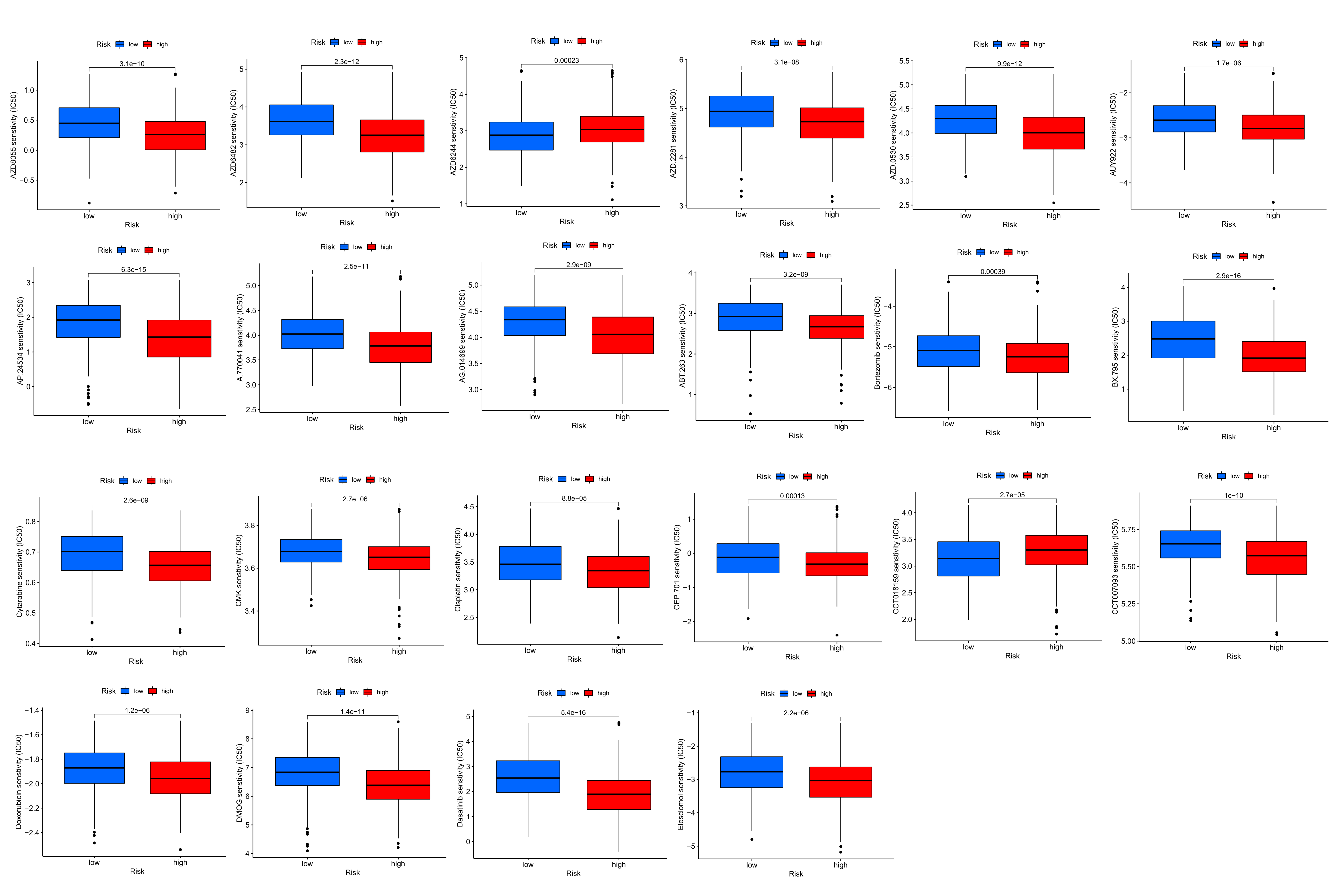

Supplement: Supplementary file 5 [file Image4.tif]
